# Demographics, Tumor Biology and Treatment Outcomes of Male Breast Cancer Patients From a Tertiary Cancer Center in North India: A Real-World Study

**DOI:** 10.7759/cureus.115580

**Published:** 2026-09-01

**Authors:** Deepak Gupta, Ajay Bapna, Anil K Gupta, Sanjeev Patni, Naresh Ledwani, Salila Chandorkar

**Affiliations:** 1 Medical Oncology, Bhagwan Mahaveer Cancer Hospital and Research Centre, Jaipur, IND; 2 Surgical Oncology, Bhagwan Mahaveer Cancer Hospital and Research Centre, Jaipur, IND

**Keywords:** hormone receptor positive, male breast cancer, malignancy, metastatic breast cancer, treatment

## Abstract

Background: Male breast cancer (MBC) is a rare malignancy, and treatment recommendations are largely extrapolated from female breast cancer data. Real-world evidence from India remains limited.

Objective: The objective of this study is to evaluate the demographic, clinicopathological, treatment, and survival characteristics of MBC patients treated at a tertiary cancer center in India.

Methods: This retrospective real-world study included MBC patients treated between January 2012 and January 2024. Demographic, clinicopathological, treatment, and outcome data were analyzed using descriptive statistics and Kaplan-Meier survival estimates.

Results: A total of 107 patients were included. The median age at diagnosis was 62 years (range: 26-82). Left-sided disease was slightly more common (51.4%), and invasive ductal carcinoma was the predominant histology (88.8%). Eighteen patients (16.8%) presented with advanced/metastatic disease. Hormone receptor positivity was high, with estrogen receptor, progesterone receptor, and human epidermal growth factor receptor 2 positivity rates of 88.5%, 89.1%, and 7.7%, respectively. Modified radical mastectomy was the most common surgical procedure (75.6%). At a median follow-up of 27.9 months, the median overall survival was 111.7 months.

Conclusion: MBC in this study was characterized by a substantial proportion of patients presenting with advanced disease and high hormone receptor positivity. Greater awareness and earlier diagnosis may improve outcomes in this rare malignancy.

## Introduction

Male breast cancer (MBC) is a rare malignancy, accounting for approximately 0.5-1% of all breast cancer cases worldwide. In India, MBC represents less than 1% of total breast cancer cases, reflecting global trends [1]. Data derived from the National Cancer Registry Programme (NCRP) and various multicentre assessments indicate that the incidence of MBC in India is slowly rising, with recent data showing a 20-25% increase over the past 10-15 years likely due to improved diagnostic facilities, greater awareness, and enhanced reporting practices [2,3].

MBC predominantly affects older men, with a median age at diagnosis ranging from 60 to 70 years. The majority of tumors are estrogen receptor (ER) positive, followed by progesterone receptor (PR) positive and human epidermal growth factor receptor 2 (HER2) positive [4-7]. Established risk factors for MBC include a positive family history, elevated estrogen or prolactin levels, and genetic mutations, particularly BRCA2 mutations, which are more commonly associated with MBC than BRCA1 mutations. Additional risk factors include obesity, alcohol consumption, chronic liver disease, and Klinefelter syndrome [8].

The prognosis of MBC is primarily influenced by the stage at diagnosis, tumor size, lymph node involvement, and the presence of distant metastases. Due to the rarity of the disease and the consequent lack of prospective clinical trial data, the management of MBC is largely extrapolated from treatment guidelines established for female breast cancer. Current treatment strategies generally consist of surgery, chemotherapy, radiotherapy, endocrine therapy, and targeted therapy, as indicated [9-11].

Despite these advances, there remains a marked paucity of Indian data on MBC. Therefore, this study was conducted to evaluate the demographic profile, tumor characteristics, treatment patterns, and outcomes of patients with MBC treated at our center. By doing so, this study aims to contribute to the limited existing Indian literature and help inform future management approaches for this rare malignancy.

## Materials and methods

This retrospective, real-world, and investigator-initiated study was conducted at Bhagwan Mahaveer Cancer Hospital & Research Centre (BMCHRC), Jaipur, India. The study was approved by the Institutional Ethics Committee of BMCHRC (BMH/2026/705). Male patients with a confirmed diagnosis of breast cancer and diagnosed from January 2012 to January 2024 were included in the study. All MBC patients who had a confirmed histopathological diagnosis were included in that period. As this study was a retrospective, real-world study, a formal sample size was not calculated. All patients registered with the hospital registry as breast cancer patients and male as gender were analyzed, but only those patients with a confirmed histopathological diagnosis were included. A total of 107 patients were included in the analysis. Baseline demographics and clinical profile variables evaluated included age, hormone receptor status, laterality, sequence of treatment, and clinical and composite staging. Overall survival (OS) at the last recorded follow-up was noted, and progression-free survival (PFS) could not be evaluated as dates of progression were not available. Data were entered into MS Excel and validated. Statistical analysis was performed using IBM SPSS Statistics for Windows, Version 27 (Released 2019; IBM Corp., Armonk, New York, United States). Descriptive statistics (mean, median, and percentages) were used to summarize baseline characteristics. Survival outcomes were evaluated using the Kaplan-Meier method.

## Results

A total of 107 male patients had a confirmed diagnosis of breast cancer between January 2012 and January 2024. The median age (range) at diagnosis was 62 (26-82) years. Breast lump was the most common presenting complaint, with a median duration (range) of 4 (0.5-80) months. The left breast was slightly more frequently involved, accounting for 51.4% of cases. Ductal carcinoma was the predominant histologic subtype, observed in 88.8% of patients, whereas papillary carcinoma accounted for 3.7%, followed by mucinous and lobular carcinomas, each comprising 2.8% of cases. ER, PR, and HER2 positivity rates were 88.5%, 89.1%, and 7.7%, respectively. At presentation, stage III disease was most frequent (40.7%), followed by stage I (27.1%), stage IV (19.8%), and stage II (12.3%), respectively. Lymphovascular and perineural invasion were present in 27.1% and 12.1% of patients, respectively (Table 1).

**Table 1:** Patient and disease characteristics Data are presented as n (%) unless otherwise specified. The variation in denominators arises because not all variables were available or assessed for all 107 patients. For each characteristic, the number of evaluable patients (n) is specified alongside the variable. Therefore, the counts within each category are based on the respective subgroup denominator and are not expected to sum to the total cohort of 107 patients. ER: Estrogen receptor; PR: progesterone receptor; HER2: human epidermal growth factor receptor 2

Patient characteristics	
Median (range) age at diagnosis (years)	62 (26–82)
Median (range) duration of lump (months)	4 (0.5–80)
Laterality (n=105)	
Right	51 (48.6)
Left	54 (51.4)
Histology (n=107)	
Ductal	95 (88.8)
Papillary	4 (3.7)
Lobular	3 (2.8)
Mucinous	3 (2.8)
Cribriform	1 (0.9)
Medullary	1 (0.9)
Receptor status	
ER positive (n=96)	85 (88.5)
PR positive (n=92)	82 (89.1)
HER-2 positive (n=91)	7 (7.7)
T stage (n=69)	
0	1 (1.4)
1	4 (5.8)
2	30 (43.4)
3	3 (4.3)
4	31 (44.9)
N stage (n=67)	
0	27 (40.3)
1	26 (38.8)
2	13 (19.4)
3	1 (1.5)
Clinical stage (n=81)	
1	22 (27.1)
2	10 (12.3)
3	33 (40.7)
4	16 (19.8)
Pathological stage (n=71)	
1	8 (11.3)
2	36 (50.7)
3	25 (35.2)
4	2 (2.8)
Grade (n=69)	
1	16 (23.2)
2	39 (56.5)
3	14 (20.3)
Lymphovascular invasion (LVI) (n=107)	29 (27.1)
Perineural invasion (PNI) (n=107)	13 (12.1)

Metastasis was reported in 16.8% (18/107) of MBC patients, and the common site of metastasis was bone (21.1%, 4/19) followed by lung (15.8%, 3/19). The majority of patients had multiple sites of metastasis (Table 2).

**Table 2:** Metastatic disease status and sites *The data for metastasis was evaluated in 92 patients out of 107. The denominator was 107 for % calculation. #One patient may have multiple sites of metastasis. The denominator was 19 for % calculation.

Metastatic disease (n=107*), n (%)	
Yes	18 (16.8)
No	69 (64.5)
Not available	15 (14.0)
Metastatic site (n=19^#^), n (%)	
Bone	4 (21.1)
Lung	3 (15.8)
Liver	1 (5.3)
Scalp	1 (5.3)
Brain	1 (5.3)
Multiple	9 (47.4)

Modified radical mastectomy (MRM) was the most common surgical procedure performed, which was around 75.6% (68/90) of cases (Table 3). Among those receiving adjuvant chemotherapy, the most used regimens were EC/T (27.9%, 19/68), AC/T (20.6%, 14/68), and EC (13.2%, 9/68), followed by TC (4.4%, 3/68), respectively. Adjuvant hormonal therapy and radiotherapy were administered to 86.9% (53/61) and 49.5% (53/107) of patients, respectively (Table 3).

**Table 3:** Treatment characteristics and details Percentages were calculated by taking the respective ‘n’ as the denominator for each category. The sum of patients across treatment categories exceeds/does not correspond to the total cohort (n = 107), as patients could receive multiple treatment modalities; hence, these categories are not mutually exclusive. MRM: Modified Radical Mastectomy; BCS: Breast Conserving Surgery; HT: Hormonal Therapy; CT: Chemotherapy; NACT: Neoadjuvant chemotherapy; AC/T: Doxorubicin + Cyclophosphamide followed by Paclitaxel; EC: Epirubicin + Cyclophosphamide; EC/T: Epirubicin + Cyclophosphamide followed by Paclitaxel/Docetaxel; TC: Docetaxel + Cyclophosphamide

First-line treatment received (n=90), n (%)	
MRM	68 (75.6)
BCS	2 (2.2)
NACT	9 (10.0)
HT	6 (6.7)
CT	5 (5.6)
Adjuvant chemotherapy (n=68), n (%)	
EC/T	19 (27.9)
AC/T	14 (20.6)
EC	9 (13.2)
TC	3 (4.4)
Adjuvant hormonal therapy (n=61), n (%)	53 (86.9)
Adjuvant radiotherapy (n=107), n (%)	53 (49.5)

At a median (range) follow-up of 27.9 (0-143.5) months, the median OS was 111.7 months. Being a real-world study, PFS could not be evaluated, as dates of progression were not available (Figure 1). Three-year OS probability was 0.834 (95% CI: 0.727-0.901) and five-year OS probability was 0.774 (95% CI: 0.652-0.858). 

**Figure 1:**
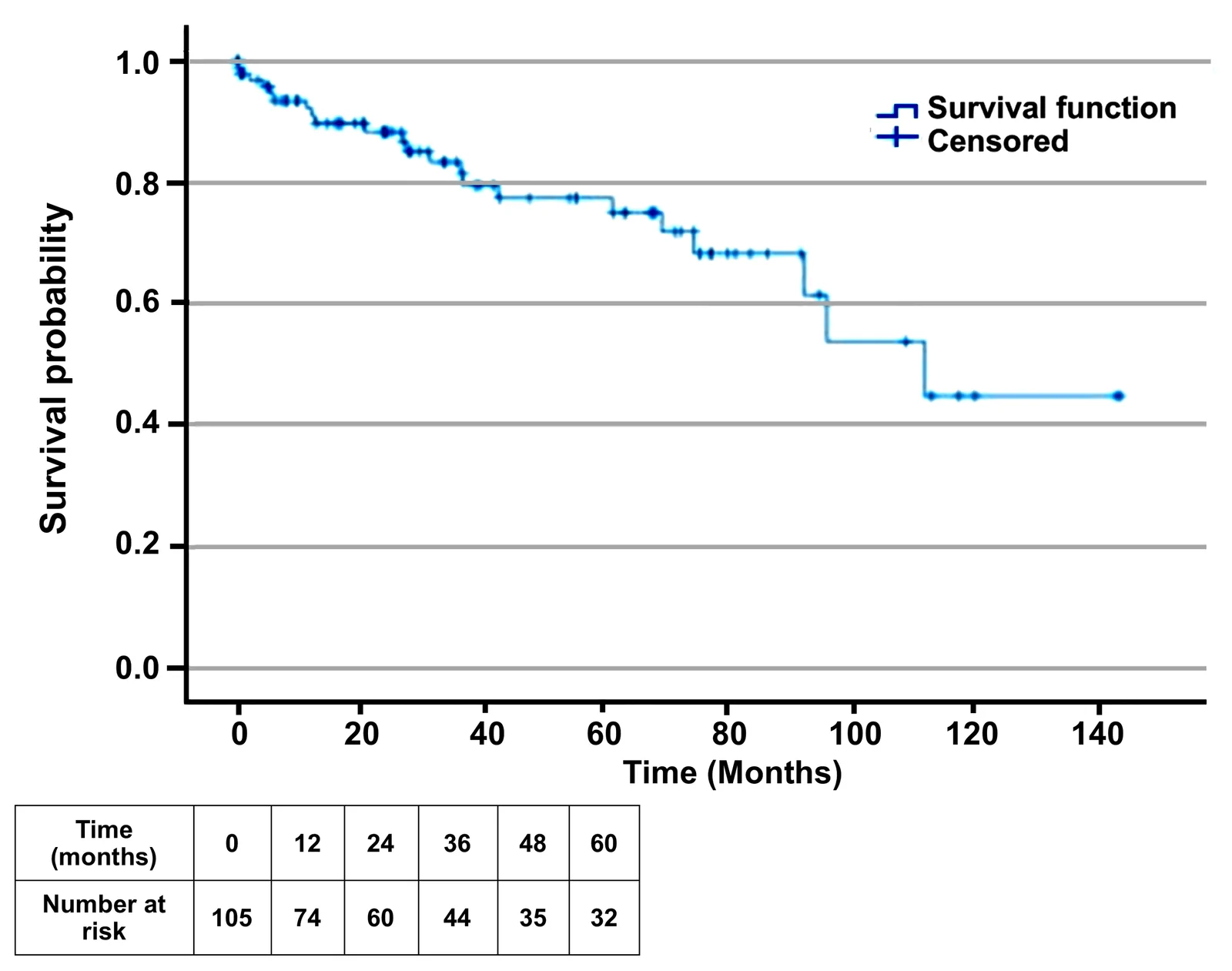
Kaplan-Meier survival curve showing overall survival for all patients (N=107) Three-year overall survival probability was 0.834 (95% CI: 0.727 to 0.901) while five-year overall survival probability was 0.774 (95% CI: 0.652 to 0.858).

## Discussion

MBC is a rare clinical entity, and evidence guiding its management is largely extrapolated from female breast cancer studies. In this retrospective study of 107 male patients, we evaluated demographic characteristics, tumor biology, treatment patterns, and survival outcomes, providing important real‑world insights from an Indian tertiary cancer center.

Table 4 represents the comparison of the present study with previous studies. In the present study, the median age at diagnosis was 62 years, which is consistent with findings reported by Khandelwal et al. (62.5 years) and Potu et al. (60 years) [12,13]. This uniformity across studies indicates that MBC predominantly affects men of this age group, reaffirming age as a consistent epidemiological feature in the Indian population. A similar age distribution has been reported in the multicentric series by Chhabra et al. [14], where the median age was 59 years, further supporting that Indian MBC patients tend to present slightly earlier compared to Western data.

**Table 4:** Comparison of the present study with previous studies ER: Estrogen Receptor; PR: Progesterone Receptor; HER2: Human Epidermal Growth Factor Receptor 2; TNBC: Triple-Negative Breast Cancer

Authors	Study Period	Study Design	No. of Patients	Median Age (Years)	Follow-up (Months)	Hormone Profile	Factors Affecting Outcome
Present study	2012–2024	Retrospective (single-center)	107	62	27.9	ER 88.5%, PR 89.1%, HER2 7.7%	Advanced stage, delayed presentation, nodal involvement
Khandelwal et al. (2021) [12]	2015–2019	Retrospective observational (case series)	34	62.5	16.5	ER 76.5%, PR 73.5%, HER2 2.9%, TNBC 17.6%	High metastatic burden, delayed presentation
Potu et al. (2026) [13]	2019–2025	Retrospective	15	60	NA	HR+ 80%, HER2+ 40%, triple positive 26.7%	Advanced stage, nodal involvement, delayed diagnosis
Chhabra et al. (2019) [14]	2010–2017	Retrospective multicenter case series	106	59	NA	ER 81%, PR 76%, HER2 25%	Delay in presentation, stage at diagnosis
Pawar et al. (2021) [15]	2016–2021	Retrospective observational (single-center)	16	NA	NA	ER/PR+: 12 pts, HER2+: 4 pts, TNBC: 2 pts	Late-stage presentation, diagnostic delay
Soni et al. (2023) [16]	1991–2020	Retrospective cohort	106	57	60	ER 41.5%, PR 25.5%, HER2 8.5%, TNBC 20.8%	Long symptom duration, advanced stage, nodal positivity, treatment modality

The median duration of symptoms in our study was four months, which is slightly shorter than the six‑month delay observed in other published studies [12,13]. Despite this relatively shorter duration, a large proportion of patients in our study presented with advanced disease. Similar observations have been reported by Pawar et al. [15], where delayed diagnosis contributed to advanced-stage presentation. These findings suggest that even modest delays in seeking medical attention can result in significant disease progression, highlighting the aggressive nature of MBC and the lack of awareness among patients. In contrast, Soni et al. [16] reported a markedly longer mean duration of symptoms (26.2 months), indicating that delayed health-seeking behavior remains a major concern in certain populations and significantly contributes to late-stage presentation.

Histopathologically, invasive ductal carcinoma was the predominant subtype in our study, accounting for 88.8% of cases. This finding aligns with previously cited studies [12,13,15], where ductal carcinoma was the most common histological type. The occurrence of less common variants such as papillary and mucinous carcinoma in our study is also in agreement with previously reported data [12,13,15], confirming the limited histological diversity of MBC. Comparable findings have been documented by both Chhabra et al. (93%) [14] and Soni et al. [16] (90.5%), reinforcing that infiltrating ductal carcinoma overwhelmingly predominates in MBC across Indian studies.

The hormone receptor profile observed in our study showed a high ER (88.5%) and PR (89.1%) positivity, which is comparable to previously published literature studies [12,13]. These observations confirm that MBC is largely hormone-driven and responsive to endocrine therapy. However, HER2 positivity in our study (7.7%) was notably lower than that reported by Potu et al. (40%) [13], while being closer to the rates observed by Khandelwal et al. [12] (2.9%). This variability may reflect biological heterogeneity or differences in testing practices but overall supports the understanding that HER2 amplification is less frequent in MBC compared to hormone receptor positivity. Supporting this, Chhabra et al. [14] reported ER/PR positivity rates of 81% and 76% respectively, with HER2 positivity of 25%, while Soni et al. [16] also observed relatively lower HER2 expression (8.5%), highlighting variability but consistent dominance of hormone receptor positivity across studies.

In our study, more than 60% of patients presented with stage III or IV disease, which is comparable to the findings of Potu et al. [13], where 86.7% of patients had advanced-stage disease, and Khandelwal et al. [12], who reported metastatic presentation in nearly half of the study. Pawar et al. [15] similarly identified late-stage disease as a dominant clinical feature. This consistent trend is one of the most critical determinants of prognosis in MBC. The study by Soni et al. [16] also demonstrated a high proportion of locally advanced and metastatic disease, with stage III and IV disease accounting for a significant majority of cases, further emphasizing delayed diagnosis as a persistent challenge in India.

Metastatic disease at presentation was observed in 21.5% of patients in our study, which, although lower than the 47.1% reported by Khandelwal et al. [12], still represents a substantial disease burden. A comparable metastatic disease burden of 19.8% was observed by Soni et al. [16], indicating that a considerable proportion of patients present with distant disease at diagnosis.

In terms of treatment, MRM was the most frequently performed surgical procedure in our study, reflecting its established role as the standard of care in MBC. Breast-conserving surgery was performed less frequently, primarily due to advanced stage and tumor characteristics at presentation. This observation is in line with the findings of Pawar et al. [15], where late-stage disease limited the feasibility of conservative approaches. Furthermore, the high utilization of hormonal therapy in our study corresponds with the high hormone receptor positivity, consistent with trends reported in other studies [12,13]. Similar treatment patterns were observed in Chhabra et al. [14], where MRM was performed in the vast majority (92%) of patients, and in the study by Soni et al. [16], where radical surgery with multimodality treatment including radiotherapy and systemic therapy constituted the standard approach.

Across all studies, including ours, certain prognostic factors emerge consistently. Advanced stage at diagnosis, nodal involvement, presence of metastasis, and delay in presentation are repeatedly identified as key determinants of poor outcome. Khandelwal et al. [12] and Potu et al. [13] emphasized the adverse impact of delayed diagnosis and metastatic disease, while Pawar et al. [15] highlighted late-stage presentation as a major contributor to prognosis. This is further supported by Soni et al. [16], who demonstrated improved survival outcomes with appropriate multimodality treatment, particularly with the use of radiotherapy and hormonal therapy in suitable patients. Despite a significant proportion of advanced-stage cases in our study, the relatively favorable overall survival may be attributed to the high prevalence of hormone receptor-positive disease and the consequent use of endocrine therapy.

Our findings are also consistent with observations from large international databases and contemporary guideline recommendations. Analyses from the Surveillance, Epidemiology, and End Results (SEER) program have demonstrated that MBC patients are typically diagnosed at an older age and more frequently present with larger tumors and nodal involvement compared with female breast cancer patients, while exhibiting substantially higher rates of hormone receptor positivity and lower rates of hormone receptor-negative disease [17,18]. Similarly, National Cancer Database (NCDB) studies involving more than 24,000 patients reported a mean age at diagnosis of approximately 66 years, ER positivity of 88.5%, PR positivity of 79.6%, HER2 positivity of 7.9%, and a higher proportion of stage IV disease at presentation, findings remarkably similar to those observed in our cohort [19]. These parallels suggest that the biological profile of MBC in Indian patients closely resembles that reported in Western populations, particularly with respect to the predominance of hormone receptor-positive disease. Furthermore, contemporary international guidelines from the American Society for Clinical Oncology (ASCO), the European Society for Medical Oncology (ESMO), and the National Comprehensive Cancer Network (NCCN) recommend that the management of MBC largely follows treatment principles established for female breast cancer because of the scarcity of prospective male-specific clinical trials [20-22]. The concordance between our findings and these large international datasets reinforces the external validity of our results and highlights the persistent global challenge of delayed diagnosis in MBC despite advances in treatment.

Limitations

This study has several limitations. Its retrospective single-center design and relatively small sample size may limit generalizability and introduce selection bias. Missing and incomplete data from physical medical records, treatment heterogeneity, and primarily descriptive statistical analyses restricted detailed outcome assessment. Information on recurrence, progression, genetic factors, and family history was limited, and changes in staging and treatment practices over the study period may have influenced results. Despite these limitations, this study provides valuable real-world evidence and adds to the limited data on MBC in India.

## Conclusions

MBC is characterized by a uniform pattern of older age at presentation, high hormone receptor positivity, and a predominance of advanced-stage disease at diagnosis. While the biological profile offers opportunities for targeted treatment, delayed diagnosis and high tumor burden continue to be the most significant challenges, underlining the need for improved awareness and early detection in men.
